# Evaluation of typical ecosystem services in Dabie Mountain area and its application in improving residents' well-being

**DOI:** 10.3389/fpls.2023.1195644

**Published:** 2023-06-06

**Authors:** Muyi Huang, Guozhao Zhang, Qilong Wang, Qi Yin, Jizhong Wang, Weihua Li, Shaoru Feng, Qiaojun Ke, Qin Guo

**Affiliations:** ^1^ School of Environment and Energy Engineering, Anhui Jianzhu University, Hefei, Anhui, China; ^2^ Anhui Provincial Key Laboratory of Environmental Pollution Control and Resource Reuse, Hefei, Anhui, China; ^3^ College of Management, Sichuan Agricultural University, Chengdu, Sichuan, China; ^4^ Guangzhou (GRG) Metrology & Test (Hefei) CO., Ltd, Hefei, Anhui, China; ^5^ School of Architecture and Urban Planning, Anhui Jianzhu University, Hefei, Anhui, China

**Keywords:** ecosystem services, multidimensional well-being, coupling coordinated development degree, geographical detector, Dabie Mountain area

## Abstract

Research on ecosystem services and residents’ well-being in old revolutionary base areas is an important task for China’s ecological civilization construction and rural revitalization. Taking Jinzhai County, the core area of Dabie Mountains, China, as an example, based on InVEST model, the methods of spatial autocorrelation and coupling coordinated development degree, the spatiotemporal evolution, spatial heterogeneity and coupling association patterns of ecosystem services and multidimensional well-being in the study area from 2005 to 2020 were discussed. The major results are: In the past 15 years, in the core area of the Dabie Mountains, ecosystem services such as food supply, soil retention and water yield showed an upward trend, carbon sequestration and biodiversity maintenance showed a downward trend. The comprehensive index of multidimensional well-being in the core area of Dabie Mountain increased by 27.23% and the spatial difference in multidimensional well-being is gradually narrowing. By the analysis of coupling coordination, the number of units with the type of coupling disharmony between ecosystem services and multidimensional well-being in the study area decreased significantly from 56.85% in 2005 to 26.81% in 2020, respectively. The analysis of geographical detection showed that the habitat quality factor was the dominant controlling factor of coupling coordination spatial difference. By bivariate spatial autocorrelation analysis, in the past 15 years, the number of units with the “high ecology-high well-being” synergy type increased from 5.44% to 13.31%. The results can provide a reference for accurate identification, optimal regulation and synergistic improvement between ecosystem services and relative poverty in the Dabie Mountain area.

## 1 Introduction

Research on ecosystem services provides a comprehensive practical approach and an important way to address the environmental issues included in global sustainable development (Guerry et al., 2015; Ouyang et al., 2016), and it also provides an important practice field for the research on the core proposition of man-land relationship in geography (Li et al., 2014; Zhao et al., 2018). Ecosystem services are the link between ecosystem and human well-being, boosting the research on the spatial association between ecosystem services and residents’ well-being is conducive to ecosystem management and control, as well as provide an important theoretical basis for the “win-win” decision-making of the coordinated improvement of ecological quality and human well-being (Jim and Chen, 2009; Dai et al., 2016; Zheng et al., 2019). As an important research field about man-land coupled system, the relationship between ecosystem services and human well-being is the hotspot and frontier of current research, and related research has made great progress in methods, ideas and frameworks, however, a unified research model has not yet been formed. Among them, Haines-Young et al. (Haines-Young and Potschin, 2010) proposed a conceptual model of ecosystem cascade linking ecological processes and elements of human well-being, which is helpful to understand the general process from service formation to human well-being. It was also conducive to distinguishing the well-being components of ecosystems (Li et al., 2013; La Nottea et al., 2017). Scholars at home and abroad have carried out a considerable amount of research on this (Fedele et al., 2017; Robert et al., 2017; Benedetto et al., 2019), such as Fedele et al. (Fedele et al., 2017). have optimized the cascade framework and discussed the mediating factors and human regulation mechanism of the ecosystem services transfer process. Li et al. (Li et al., 2014) proposed the research categories and the subject domains of different links in the cascade framework. Based on the conceptual model of the cascade framework, Fu et al. (Fu et al., 2017) established an indicator system for assessing biodiversity and ecosystems in China. Relevant research has important reference value for in-depth discussion of ecosystem final service assessment and human well-being. Currently, research is increasingly moving from single issues such as conceptual description, assessment of ecosystem services and human well-being to comprehensive analysis from the perspective of sustainable development (Huang et al., 2016). Among them, there are ample research results related to ecosystem services and human well-being, and the objects cover the Loess Plateau (Li and Zhou, 2016), natural reserve (Wu et al., 2015), grassland (Han et al., 2018), basin (Xu et al., 2019), ocean (David et al., 2020), arid area and so on (Hu et al., 2022a). In recent years, the relationship between ecosystem services and the livelihood of farmers, the well-being of residents in poverty-stricken areas has attracted widespread attention from the academic community. Relevant research mainly conducts mutual analysis by constructing different service indicators, statistics, and livelihood or well-being indicators of questionnaires. The research methods mainly include matching statistics (Suich et al., 2015), regression analysis (Hu et al., 2018), coupling coordinated degree (Pan et al., 2020) and perception questionnaire (Yang et al., 2019; Marcinkevičiūtė et al., 2022; Xiong et al., 2022), etc. The above research has played a good role in complementing and promoting the understanding of the relationship between ecosystem services and human well-being at different space-time scales, but the impact mechanism, coupling model, ecological process of them still needs to be explored (Zang and Zou, 2016; Zhao, 2017). Strengthening the understanding of the process of ecological services affecting well-being will be beneficial to ecological optimization and management decision-making. According to the development characteristics of the study area, Xu et al. (Xu et al., 2019), based on the perspective of the carbon flow process dominated by human activities, discussed the relationship between ecological services and human well-being as well as the optimization strategies of Manas Basin in Xinjiang.

At present, the spatiotemporal coupling mechanism between ecosystem services which has externalities and scale effects, and multi-level well-being needs to be deepened (Carpenter et al., 2009). For example, Qiao et al. (Qiao et al., 2017) used the Granger causality method to explain the causal relationship between the spillover effects of ecosystem services in the river basin and the well-being of residents. Yang et al. (Yang et al., 2021) discussed the quantitative relationship between ecosystem services and human well-being. Fu et al. (Fu L. et al., 2022) used the SEM method to analyze the relationship between ecosystem services and rural residential well-being in the Xin’an River Basin, China. Qiu et al. (Qiu et al., 2022) established a model based on SEM to study the influential paths of ecosystem services on human well-being in the context of sustainable development goals. Therefore, combining econometric methods with other methods to strengthen the exploration of the coupling coordination characteristics, association patterns, and spatial heterogeneity of ecosystem services and human well-being will contribute to promoting the understanding of the process of synergistic improvement between the two.

In 2020, China achieved a comprehensive victory in the battle against poverty, and the issue of relative poverty governance in the post-poverty era is becoming the focus of tasks. To further consolidate the achievements of poverty alleviation, effectively connect rural revitalization, and help solve the problem of relative poverty with ecological and economic development, it is of great significance for the sustainable development of former revolutionary base areas, areas inhabited by minority nationalities, remote and border areas and poverty-stricken areas. Carrying out theoretical and empirical research on ecosystem services and residents’ well-being also provides a theoretical basis and solutions for the accurate identification, optimization regulation and policy formulation of “ecology-well-being” issues (Huang et al., 2019a). The Dabie Mountain area, as a significant ecological functional area and a former revolutionary base area in China, conducting further research on the correlation between ecosystem services and the well-being of residents aligns with the national objective of promoting ecological civilization and implementing rural revitalization strategies in former revolutionary base areas. Taking Jinzhai County, the former revolutionary base areas in Dabie Mountains, as an example, we carried out research on the spatiotemporal pattern, coupling association, spatial difference characteristics of typical ecosystem services and residents’ well-being, analyzed the evolution of ecosystem services in former revolutionary base areas, and discussed the coupling coordination development degree between ecosystem services and residents’ well-being, revealed the association pattern and spatial heterogeneity of the two by applying a combination of approaches with multisource data. The research results can provide a certain theoretical basis for the “win-win” target policy of the coordinated development of ecosystem services and residents’ well-being in the Dabie Mountain area (Huang et al., 2019a).

## 2 Materials and methods

### 2.1 Study area

**Figure 1:**
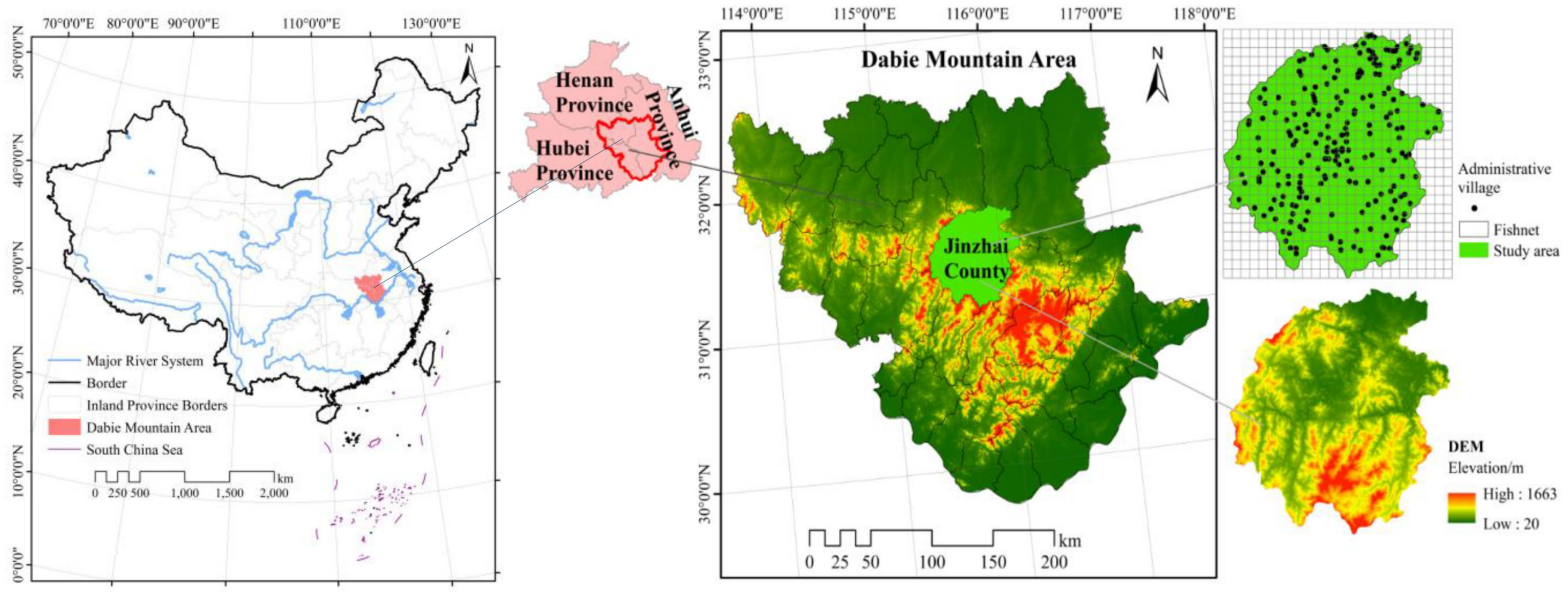
Study area.

This study takes Jinzhai County, the core area of the Dabie Mountains, China, as the research region. Jinzhai County, located between 31°06′~ 31°48′N and 115°22′~ 116°11′E, with a total area of 3814 km^2^, is the largest mountainous county and tourism resource county with the largest area and population in Anhui Province of China. It is located in the hinterland of Dabie Mountains and is the core area of the junction of Hubei, Henan and Anhui, and the western region of Anhui Province, as shown in **Figure 1**. It is also the second largest “General County” in China and the former revolutionary base area, known as “the cradle of the Chinese Red Army, the hometown of generals”. In December 2019, Jinzhai County became a pilot area for the construction of the national rural governance system. In April 2020, Jinzhai County exited from the poverty county sequence. Jinzhai County is located in the core area of Dabie Mountains whose terrain descends from southwest to northeast. Dabie Mountains run through the whole territory which is characterized by undulating mountains, crisscross rivers and abundant water resources, from southwest to northeast.

### 2.2 Data source and processing

The used data in this study comprises various multi-source data types, including spatial data, statistics data and questionnaires data. The land use data, which has a spatial resolution of 30 meters in 2005 and 2020, was collected, along with DEM data (SRTM digital elevation data in GRID format with a 30 m spatial resolution); soil data (Chinese soil type distribution map, Chinese soil data set HWSD_China_Subset_v1.1); meteorological data (extracted from the interpolation map based on ArcGIS 10.2 with the meteorological element observation data of 44 meteorological stations in the study area and surrounding areas), and evapotranspiration data in 2005 and 2020. The above data are from the Chinese Academy of Sciences Resource and Environmental Science Data Center (http://www.resdc.cn/) and the Geographic Remote Sensing Ecology Network (http://www.gisrs.cn). The Normalized Difference Vegetation Index (NDVI) data was extracted from remote sensing images of Geospatial Data Cloud (http://www.gscloud.cn/), the slope data was extracted from DEM, the land use degree and human impact index factors were calculated with reference to relevant literature (Zhuang and Liu, 1997; Yang and Liu, 2022), The socio-economic statistics mainly come from the statistical yearbook and the farmer questionnaire survey. The farmers’ data come from interviews and retrospective questionnaire surveys (including village cadres and villagers) in the villages of Jinzhai County in November 2020 and August 2021. 910 questionnaires were distributed, and 856 valid questionnaires were recovered, with an effective recovery rate of 94.07%. The reliability test was conducted based on SPSS19.0. Cronbach’s Alpha coefficients were 0.950 and 0.849, both greater than 0.800. KMO values were 0.940 and 0.920, both greater than 0.800. The significance level of Bartlett’s spherical test was less than 0.001, which shows that the questionnaire design is reasonable, and the data has high validity. This paper used GPS to locate the geospatial location coordinates of each administrative village and import them into ArcGIS 10.2 to generate point shapefiles, then recorded relevant attributes of each administrative village into the point layer. The description of the data is shown in **Table 1**.

**Table 1:** Description of data.

Date type	Year	Data description	Data Source
Land use data	2005, 2020	Raster, 30 m	http://www.resdc.cn
DEM	2000	Raster, 30 m	http://www.resdc.cn
Soil data	2009	Vector, 1:10^6^	http://www.resdc.cn
Meteorological data	2005, 2020	Vector, point layer	http://www.resdc.cn
NDVI	2005, 2020	Raster, 30 m	http://www.gscloud.cn
Evapotranspiration data	2005, 2020	Raster, 30 m	http://www.gisrs.cn
Socioeconomic data	2006, 2021	/	Statistical yearbooks
Farmer questionnaires data	2005, 2020	Questionnaire	Interviews and retrospective questionnaire surveys

### 2.3 Ecosystem Services Assessment Methods

#### 2.3.1 Food supply

Food supply service is an imperative service in the agricultural ecosystem, which plays a vital role in human beings’ survival and the region’s development (Funabashi, 2018). Studies have shown that there is a significant linear relationship between yields of crop and livestock products and NDVI. Based on the land use/cover type, referring to relevant literature (Wu et al., 2017), the total yield such as food is allocated according to the ratio of the grid NDVI value to the total cultivated land NDVI value, thereby characterizing the food supply capacity of each grid. In terms of calculation methods, we allocated the output of grain according to the grid unit of cultivated land, the output of meat according to the grid unit of grassland, and the output of aquatic products according to the grid numbers of the water body. The specific methods are as follows: First, using conditional functions in ArcGIS, cultivated land, grassland, and water bodies are extracted from the land use data of the study area; Second, for cultivated land and grassland, we allocated the output based on the ratio of the NDVI value of each grid to the total NDVI value of different land types, and ultimately allocate the output of grain, meat, and milk products to the cultivated land and grassland grid units, respectively. For the allocation of aquatic products, we used an average allocation method based on the total number of water body pixels, that is, the ratio of the output of aquatic products to the total number of water grid cells to evenly allocate the number of aquatic products. The formula is as follows:


(1) Gi=Gsum×NDVIiNDVIsum

where *G_i_* refers to the food supply quantity in grid I, *G_sum_* refers to the food supply quantity of the whole area, *NDVI_i_* is the normalized difference vegetation index of grid *i*, while *NDVI_sum_* refers to the sum of NDVI values of cultivated land or grassland in the study area.

#### 2.3.2 Soil retention

By referring to relevant literature’s methods (Wang et al., 1995; William and Arnold, 1997; Cai et al., 2000; Manik et al., 2019; Li et al., 2022), the factors such as rainfall, terrain and soil are rasterized, and the grid layer and parameter table are input into the InVEST model. Then the capacity of soil retention in the study was obtained by calculating the potential amount of soil erosion (RKLS) and soil erosion (USLE) layers based on the sediment delivery ratio module in the model. The formula is as follows:


(2) $$\begin{array}{c} USLE=R\times K\times LS\times C\times P \end{array}$$

(3) $$\begin{array}{c} RKLS=R\times K\times LS \end{array}$$

(4) $$\begin{array}{c} SR=RKLS-USLE \end{array}$$

where *SR* is the quantity of soil retention (t·hm^-2^·a^-1^); *R* is the factor of rainfall erosion (MJ·mm·hm^-2^·h^-1^·a^-1^) calculated by the Wischmeier formula according to the monthly average precipitation and annual average precipitation in the study area. *K* is the factor of soil erosion(t·hm^2^·h·hm^-2^·MJ^-1^·mm^-1^); *LS* is the dimensionless slope length factor; *P* is the factor of soil and water conservation measures, between 0-1, calculated by the slope index α; *C* is the factor of vegetation coverage and crop management, between 0-1, calculated by the relationship formula between vegetation coverage and *C* value.

#### 2.3.3 Water yield

The “water yield” module in InVEST model is used to estimate the water yield. The water yield module in InVEST model is based on the Budyko framework and water balance principle (Sharp et al., 2016). InVEST model has advantages in space expression and visualization compared with other hydrological models (Leh et al., 2013; Gao and Zuo, 2021). The formula is as follows:


(5) Y(x)=(1−AET(x)P(x))·P(x)

where the *Y_(x)_* refers to the annual water yield of each grid unit *x*; *AET_(x)_* is the annual actual evapotranspiration(mm) of each grid unit *x*, while *P_(x)_* refers to the annual rainfall (mm) of each grid unit *x*.

#### 2.3.4 Carbon sequestration

The carbon storage of the ecosystem mainly includes four basic carbon pools: aboveground biomass, underground biomass, soil and dead organic matter. Ecosystem carbon storage is estimated as the storage of carbon currently stored in the landscape based on the average carbon density of carbon pools of different land use types multiplied by the area of each land use type in the area (Zhou et al., 2019; Huang et al., 2023). The carbon sequestration module in the InVEST model was used to assess the spatial distribution of carbon storage in the ecosystem of study areas in this paper. The formula is as follows:


(6) $$\begin{array}{c} C_{\_total}=C_{\_above}+C_{\_below}+C_{\_soil}+C_{\_dead} \end{array}$$

where *C__total_* refers to the total carbon storage, *C__above_* refers to the carbon storage of aboveground biomass, *C__below_* refers to the carbon storage of underground biomass, *C__soil_* refers to the carbon storage of soil carbon pool; *C__dead_* refers to the dead carbon storage of organic matter.

#### 2.3.5 Biodiversity maintenance

InVEST model calculates habitat quality by combining landscape type sensitivity and external threat intensity and evaluates biodiversity service function according to habitat quality (Peng et al., 2018b). This study calculated the habitat quality index based on “Habitat Quality” module in InVEST 3.6.0 to reflect the function of providing biodiversity services (InVEST model assumes that areas with good habitat quality have high biodiversity). The habitat quality index is a dimensionless and comprehensive index to evaluate the suitability of the regional land use types and the degree of habitat degradation. The calculation formulas and model parameter tables refer to literature (Sharp et al., 2016; Chu et al., 2018; Huang et al., 2020; Liu et al., 2022b).

#### 2.3.6 The research framework

**Figure 2:**
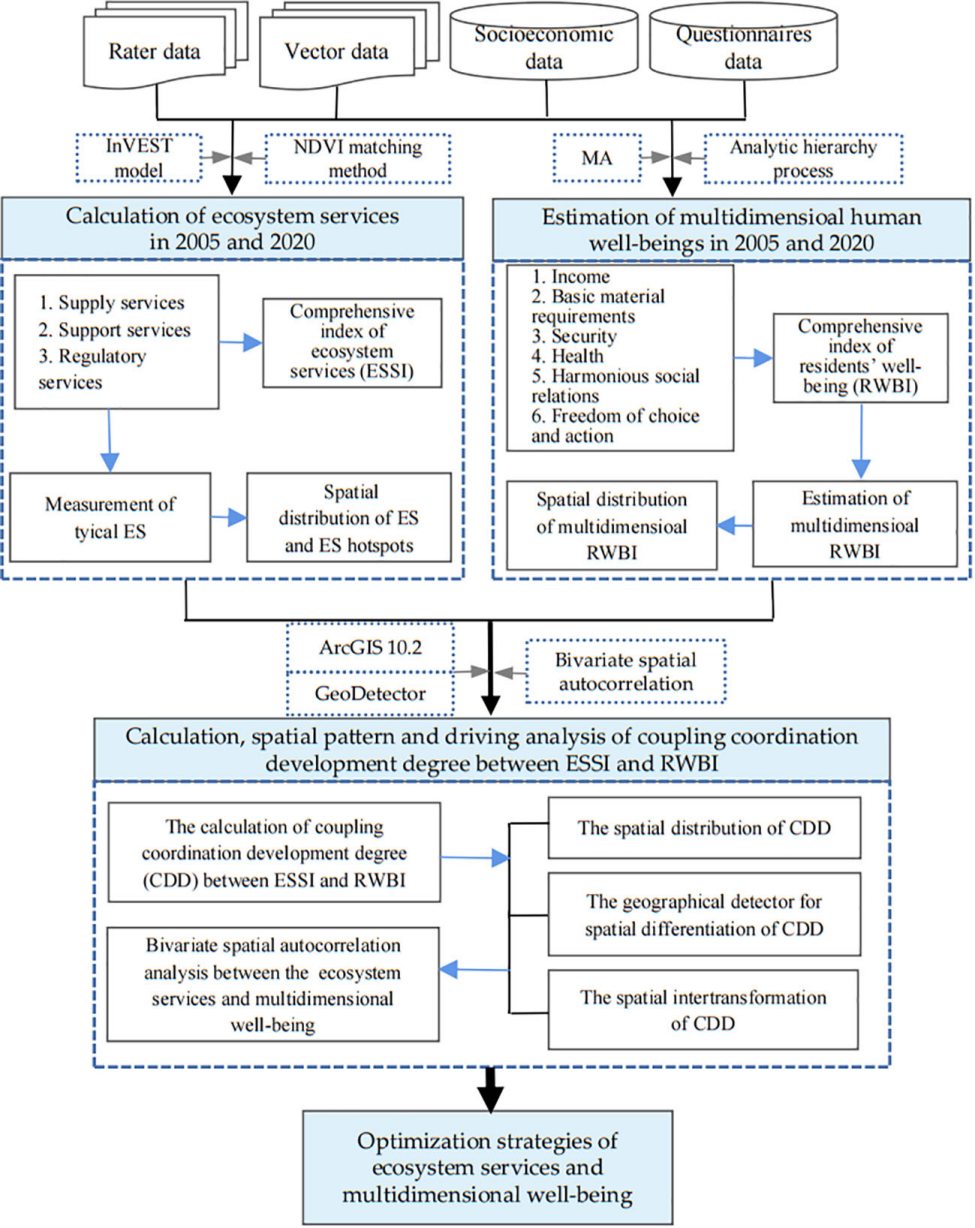
The research framework of this study.

The research framework is shown in **Figure 2**. First, based on the InVEST model and NDVI matching method, we calculated the values of the ecosystem services in 2005 and 2020. Next, based on the Millennium Ecosystem Assessment, six dimensions of parameters were selected to establish a well-being index system. Second, the comprehensive index of ecosystem services (ESSI) and the comprehensive index of residents’ well-being (RWBI) were constructed. Third, the coupling coordination development degree between the ESSI and the RWBI was calculated and analyzed. Finally, some optimization measures for coupling coordination development degree were proposed.

### 2.4 Establishment of index system and calculation of comprehensive index

#### 2.4.1 Establishment of comprehensive index system in ecosystem services and multidimensional well-being

Based on the relevant studies (Huang et al., 2019a; Huang et al., 2019b; Huang et al., 2020) and the situation of the study area, five typical ecosystem services, including food supply, soil retention, biodiversity maintenance, water yield and carbon sequestration, were selected to construct an ecological service index system; well-being index system mainly adopts the Millennium Ecosystem Assessment which is constructed by six dimensions of parameters about income (food income, income level), basic living conditions (the means of production, infrastructure), security, (resource security, personal security, from disaster), health (healthy eating, physical health), good social relations (cultural education, family relations, neighborhood relations), choice and action (work freedom, enjoy life) (Millennium Ecosystem Assessment, 2005). Through theoretical analysis and literature review (Suich et al., 2015; Zang and Zou, 2016; Chen and Chang, 2020; Xiong et al., 2022), the multidimensional well-being comprehensive index system of the Dabie Mountain area was established by selecting different dimension parameters combined with the actual situation of the study area, then the weights were determined based on the analytic hierarchy process (Gao et al., 2022). The index design scale of this study is shown in **Table 2**.

**Table 2:** The comprehensive index system of ecosystem services and multidimensional well-being.

Target layer	Rule layer	Index layer
**Comprehensive index of ecosystem services(X)**	**X_1_** supply services	**X_11_** food supply
	**X_2_** support services	**X_21_** soil retention; **X_22_** biodiversity maintenance
	**X_3_** Regulatory services	**X_31_** water yield; **X_32_** carbon sequestration
**Comprehensive index of residents’ well-being(Y)**	**Y_1_** Income	**Y_11_** per capita grain output; **Y_12_** number of livestock; **Y_13_** annual per capita income; **Y_14_** income satisfaction
	**Y_2_** Basic material requirements to maintain a high-quality life	**Y_21_** grain output per unit area; **Y_22_** per capita living area; **Y_23_** housing satisfaction; **Y_24_** water project of reconstruction; **Y_25_** toilet renovation; **Y_26_** household assets of consumer durables; **Y_27_** transportation convenience degree
	**Y_3_** Security	**Y_31_** per capita cultivated land area; **Y_32_** food safety satisfaction; **Y_33_** satisfaction of public security in the village; **Y_34_** frequency of natural disasters (landslide, drought, etc.)
	**Y_4_** Health	**Y_41_** satisfaction of vegetable consumption; **Y_42_** satisfaction of meat consumption; **Y_43_** family health; life expectancy; **Y_44_** satisfaction of medical insurance; **Y_45_** satisfaction of medical conditions in the village
	**Y_5_** Harmonious social relations	**Y_51_** per capita education level; **Y_52_** total family burden coefficient; **Y_53_** family relationship happiness degree; **Y_54_** neighborhood harmony degree
	**Y_6_** Freedom of choice and action	**Y_61_** ease of finding a job; **Y_62_** ease of affording family expenses and enjoying a happy life

#### 2.4.2 Calculation of comprehensive index in ecosystem services and multidimensional well-being

The spatial overly analysis method based on ArcGIS was used to calculate the comprehensive index of ecosystem services at the grid scale. The research data in the study area were standardized, and then the weighted average method was used to calculate the multidimensional well-being comprehensive index. The ecosystem services composite index and multidimensional well-being composite index are calculated using the following formula:


(7) $$ESSI_{i}=\sum_{j=1}^{3}W_{Xj}\cdot \sum_{j=1}^{n}W_{Xij}\cdot X_{ij}^{'}$$

(8) $$RWBI_{i}=\sum_{j=1}^{6}W_{Yj}\cdot \sum_{j=1}^{n}W_{Yij}\cdot Y_{ij}^{'}$$

where *ESSI*_i_ is the comprehensive index of ecosystem services; *RWBI*_i_ is the comprehensive index multidimensional well-being; *W_X_*_j_ and *W_Yj_* are the weights of each evaluation factor in the evaluation rule layer of ecosystem services and multidimensional well-being; *W_Xij_* and *W_Yij_* are the weights of each evaluation factor in the evaluation index layer of ecosystem services and multidimensional well-being; *X*’_ij_ and *Y*’_ij_ are the normalized value of each evaluation factor in the evaluation index layer of ecosystem services and multidimensional well-being.

### 2.5 Coupling coordination and spatial heterogeneity analysis of ecosystem services and multidimensional well-being

#### 2.5.1 Coupling coordination analysis

In order to reflect the general effectiveness of the comprehensive measurement of the research system, it is necessary to build a coupling coordinated development model to compare, analyze and evaluate the advantages and disadvantages in different dimensions (Hu et al., 2022b). This paper introduced the coupling coordinated development degree model with reference to relevant literature (Liao et al., 2020; Maimaiti et al., 2022). The formula is as follows:


(9) $$C={\left\{E\left(X\right)\cdot R\left(Y\right)/{\left(E\left(X\right)/2+R\left(Y\right)/2\right)}^{2}\right\}}^{K}$$

(10) T=α·E(X)+β·R(Y)

(11) $$D={\left(C\cdot T\right)}^{1/2}$$

where *E(X)* and *R(Y)* are the comprehensive index of ecosystem services and multidimensional well-being; *K* is the adjustment coefficient, and *K* is greater than or equal to 2 (the value here is 2); *C* is the coupling coordination degree, whose neighborhood ranges from 0 to 1, the larger *C* is, the higher coordination level will be; *T* shows that the comprehensive development index in two systems; *α* and *β* respectively represent the weights of E(X) and R(Y) in the evaluation index system, the value of each is 0.5 and the sum is 1; *D* represents the coupling coordination development degree of coupling coordination between systems, and the neighborhood of the value is 0~1. To facilitate spatial comparison and complete coupling coordination analysis at a unified scale, this paper uses the ArcGIS10.2 fishnet tool to generate 3 km grid units for services and well-being sampling (The average area of rural areas in this study area is about 17 square kilometers. In order to conduct coupling analysis between ecosystem services and residents’ well-being at a smaller unit scale and refer to relevant research literature (Huang et al., 2019a), this paper ultimately selected a 3 kilometer * 3 kilometer grid as the sample unit scale for coupling analysis). To reference relevant literature (Hu et al., 2018; Liao et al., 2020; Maimaiti et al., 2022), this paper divides the coefficient of coordinated development into five grades from low to high, namely 0≤*D*<0.2 severe disorder, 0.2≤*D*<0.4 moderate disorder, 0.4≤*D*<0.06 primary disorder, 0.6≤*D*<0.8 moderate coordination and 0.8≤*D*<1 excellent coordination.

#### 2.5.2 Bivariate spatial autocorrelation analysis

This paper adopted a bivariate spatial autocorrelation index to study the spatial association between multiple variables. Compared with the single variable spatial autocorrelation, the bivariate autocorrelation index can reveal the spatial association between different elements (Zheng et al., 2020), the formula is:


(12) $$\begin{array}{c} I_{er}^{p}=\frac{X_{e}^{p}-\bar{X_{e}}}{\sigma_{e}}\sum_{q=1}^{n}W_{pq}\frac{X_{r}^{q}-\bar{X_{r}}}{\sigma_{r}} \end{array}$$

where $I_{er}^{p}$ refers to the global spatial autocorrelation index of the bivariate (evaluation index values of items *e* and *r*) of spatial unit *p*, $X_{p}^{e}$ refers to the *e*-th evaluation index value of spatial unit *p*, $X_{r}^{q}$ refers to the *r*-th evaluation index value of spatial unit *q*; $\bar{X}$ and *σ* are the mean and variance of corresponding indicators; *W_pq_* is the spatial connection matrix between spatial units *p* and *q*.

#### 2.5.3 Research on spatial heterogeneity by geographical detector

In this paper, the GeoDetector tool was used to carry out the geographical detector of spatial heterogeneity. Its main principle is to detect the influence of independent variables on dependent variables according to the relationship between the variance within each factor layer and the total variance (Wang and Xu, 2017). *q* statistic, the value with the range [0,1], which means that the independent variable *X* explains 100×*q*% of the dependent variable *Y*, is used to measure the explanatory power of each factor to the spatial differentiation of the dependent variable in the factor detector. The formula (Wang and Xu, 2017) is:


(13) $$\begin{array}{c} q=1-\frac{1}{N\sigma^{2}}\sum_{n=1}^{L}N_{h}\sigma_{h}^{2} \end{array}$$

where *h*=1,…, *L* is the layer of the variable *Y* or the factor *X*; *N_h_* and *N* are the number of units of layer *h* and the whole area; $\sigma_{h}^{2}$ and *σ* ^2^ are the variances of *Y* in layer *h* and the whole area.

## 3 Results and analysis

### 3.1 Measurement and spatiotemporal change of the ecosystem services

#### 3.1.1 Spatiotemporal features of ecosystem services

**Figure 3:**
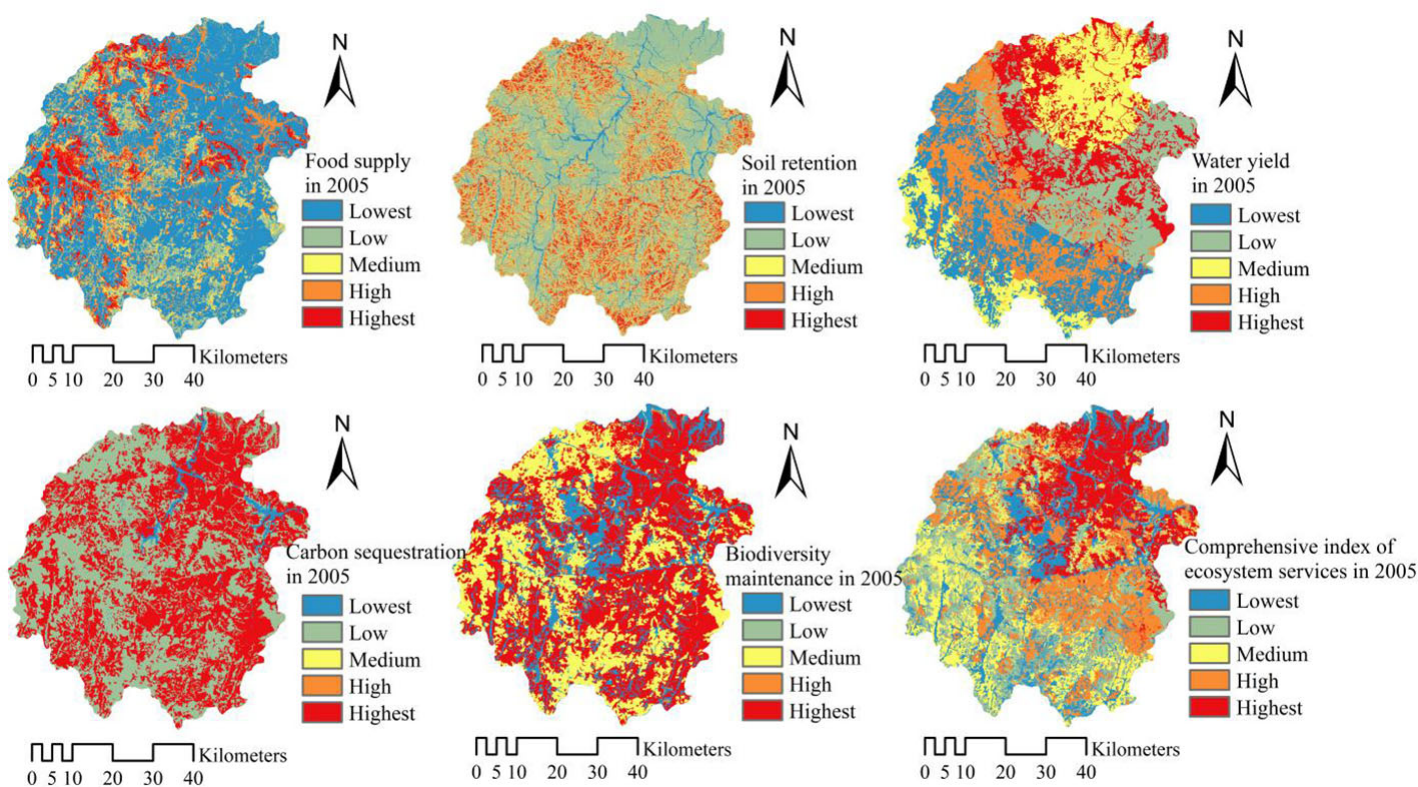
The spatial distribution of ecosystem services in the study area in 2005.

**Figure 4:**
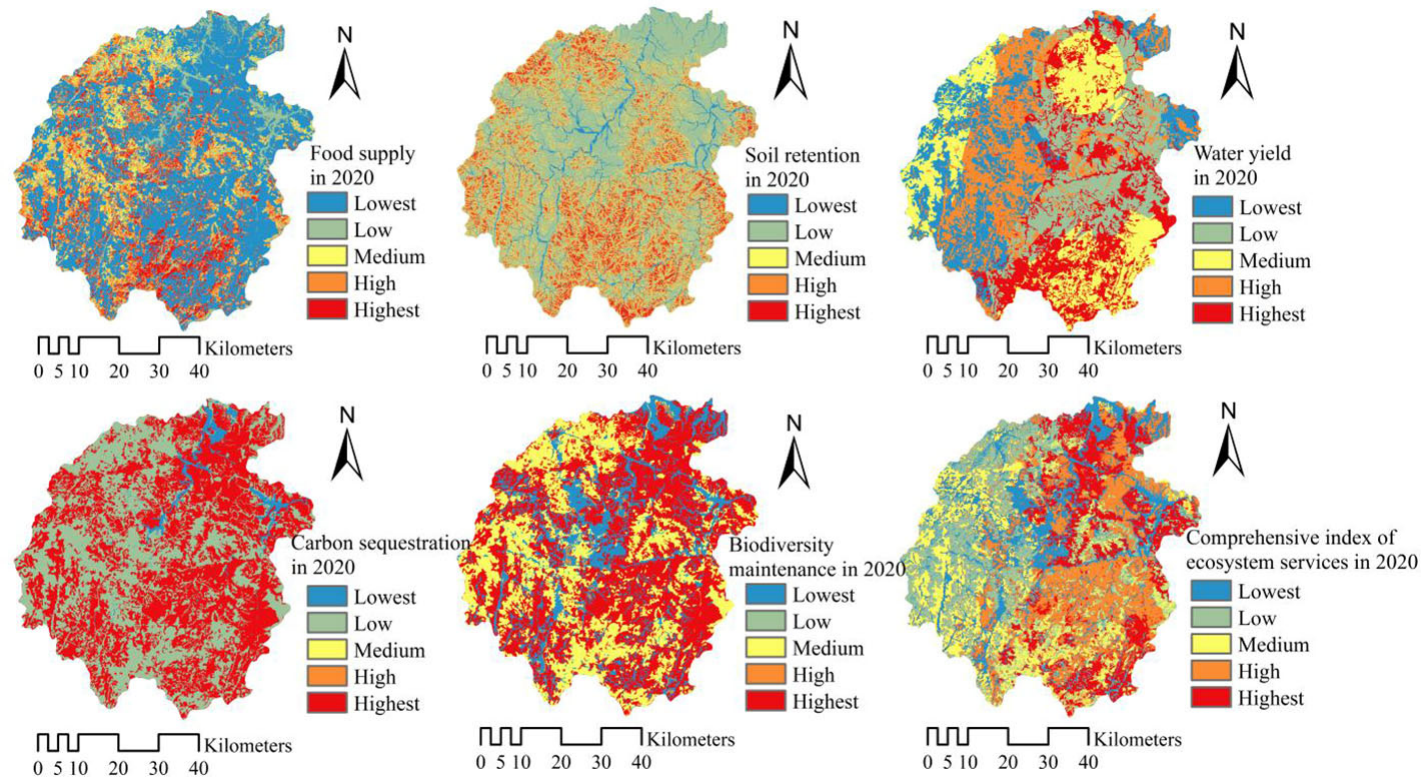
The spatial distribution of ecosystem services in the study area in 2020.

This paper obtained five typical ecosystem services and comprehensive indexes of ecosystem services each year through the calculation of relevant model methods in the core area of Dabie Mountains, including food supply, soil retention, water yield, carbon sequestration and biodiversity maintenance. The analysis showed, during 2005-2020, there was a trend of “three items rise, and two items fall”, in detail, the annual growth rates of food supply, water yield and soil retention services were 2.03%, 8.17% and 6.04%, while the annual decrease rates of carbon sequestration and biodiversity maintenance were 0.04% and 0.03%. It can be seen that in recent 15 years, water yield services showed the largest increase in the area. Generally, the comprehensive index of ecosystem services shows an increasing trend, high-level ecological service areas are mainly distributed in the northeast, and the low-value areas are scattered in the middle and southwest, as shown in **Figures 3**, **4**. In the past 15 years, high-value areas of ecosystem services in the study area showed a trend of developing toward the southeast, while low-value areas have the characteristic of gathering toward the middle. From 2005 to 2020, the statistical descriptive parameters indicated that mean values of comprehensive indexes of ecosystem services are 1.8610 and 1.9361. Thus, the changing trend of ecosystem services in the study area was to increase as a whole, and the spatial difference was more balanced in the recent 15 years.

Owing to the fact that the Dabie Mountain area was situated in the national rainfall center in 2020, water yield services increased fastest from 627.13 mm in 2005 to 1395.45 mm in 2020, with a growth of 122.52%. Meanwhile, soil retention services changed from 232.23×10^4^ t in 2005, up to 442.50×10^4^ t in 2020, with a growth of 90.54%; food supply services also showed an upward trend, from 29.25×10^4^ t in 2005, up to 38.17×10^4^ t in 2020, with the growth of 30.51%. The statistical parameters show that the standard deviation of food supply on the grid unit has risen from 0.2343 to 0.2700, which means that the spatial difference of food supply services is expanding, indicating that the planning measures such as farmland consolidation and land centralized renovation project in the core area of Dabie Mountains have played a significant role in the past 15 years. Measures such as industrial-scale operation have optimized the spatial layout of food supply carriers, thus further improving the service of food supply. In the recent 15 years, however, carbon sequestration and biodiversity maintenance showed a weak downward trend, with a decrease of about 0.50%. The analysis indicates that the typical ecosystem services in the core area of Dabie Mountains show complex changing trends during the rapid urbanization in the past 15 years. It is necessary for the local government to enhance comprehensive ecological planning and management of mountains, rivers, forests, fields, lakes and grasses, optimize ecosystem functions, then further enhance biodiversity and carbon sequestration services, and build a sound foundation for ecological progress. All of these will provide a “win-win” ecological foundation and socio-economic development conditions for rural revitalization, habitat quality improvement and relative poverty governance.

#### 3.1.2 Hotspots recognition and spatiotemporal change of ecosystem services

**Figure 5:**
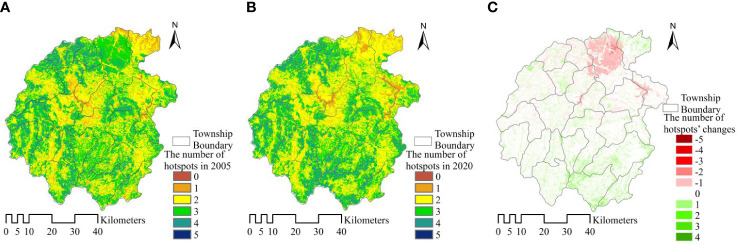
The number **(A, B)** and spatial variation of ecosystem service hotspots **(C)** in the study area from 2005 to 2020.

Based on ecological service assessment, the identification of service hotspots in the core area of Dabie Mountains is helpful to understand the strength of service supply capacity in different regions (Dade et al., 2019). Under normal circumstances, the same ecosystem can provide a variety of different supply services, but its supply capacity is considerably different (Lydia et al., 2018). Therefore, the spatial analysis method can be used to identify the grid units whose supply services of a certain type of ecosystem are greater than the average value of such services and define them as the hotspot units of dominant services, which is conducive to the precise spatial identification and control of ecological service hotspot ecological units. In this paper, The calculation of hotspot layers is mainly divided into two processes: the calculation of a single ecosystem service hotspot layer and the superposition of multiple hotspot layers, as follows: (1) based on the ArcGIS 10.2 grid calculator condition function, to calculate the hotspot layer for each ecosystem service, the average value of the ecosystem service layer was calculated, and then the raster cells above the average value were defined as hotspot areas, thus generating five ecosystem service hotspot layers respectively. (2) Based on ArcGIS 10.2 raster overlay tool, the hotspot layers of the five ecosystem services were overlaid, and the spatial hotspot distribution map of the study area was finally generated. All the units that do not exceed the average value were defined as non-hotspots, and those with one service whose value exceeds the average value were defined as Class 1 hotspots, and then 2 to 5 hotspots were defined in turn. The hotspot change map was obtained after calculating the difference between the hotspot distribution maps of the two phases. In this study, the number of hotspots in grid units has the largest change of 5 and the largest increase of 4 among the hotspot difference changes from 2005 to 2020. The spatial hotspot pattern and change distribution are shown in **Figure 5**.

Hotspot analysis shows that, in 2005, the proportion of Class 5 hotspots which has 5 hotspots is 0.16%. Successively, Class 4 hotspots account for 14.47%, Class 3 hotspots account for 36.51%, Class 2 hotspots account for 41.52%, and Class 1 hotspots account for 7.17%, non-hotspots accounts for 0.17%. Generally, service areas that have 0 to 2 hotspots account for 48.86%, and Class 3 to 5 hotspot areas account for 51.14%. While in 2020, the proportion of service areas from Class 5 to Class 1 was 0.24%, 14.69%, 33.57%, 45.69% and 5.63% respectively and non-hotspot 0.19%. Generally, services areas that have 0 to 2 hotspots account for 51.50%, Class 3 to 5 hotspots areas account for 48.50%. Contrast analysis shows that the area of high hotspots ecosystem services is shrinking, while low hotspots area is expanding, but the change is not obvious as a whole.

From the perspective of spatial changes in hotspots, the number of hotspots decreased by 5 at most and increased by 4 at most. In terms of change areas, the area that has not changed accounts for 79.20%, the second is that the number of hotspots decreased by one, accounting for 10.02% of the total area, and the number of hotspots increased by one, accounting for 9.57% of the total area, and the area with the other hotspot changes was less than 1%. The grid units with reduced hotspots are mainly distributed in Meishan Town, the northern region of the focus of social and economic development. The units with increased hotspots are mainly scattered in the whole region in space, but mainly in the southern mountains. Therefore, it is necessary to promote ecological governance, restoration, and strengthen the management and optimization of land use and ecosystems in hotspots of human activities in the core area of Dabie Mountains.

### 3.2 Characteristics of spatial distribution pattern of multidimensional well-being comprehensive index

**Figure 6:**
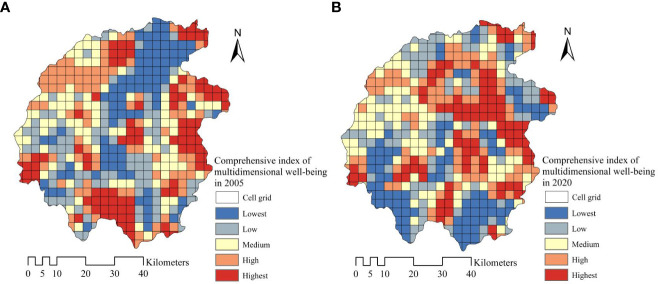
The spatial distribution of comprehensive index of multidimensional well-being in 2005 **(A)** and 2020 **(B)**.

The multidimensional well-being comprehensive indexes of Jinzhai County in the core area of Dabie Mountain in 2005 and 2020 are respectively obtained according to the formulate (8). Considering the quantitative analysis at a finer scale, this paper carried out spatial interpolation based on the IDW method, used the grid unit with a 3 km amplitude to carry out spatial statistics, and divided the well-being level into five levels, lowest, low, medium, high and highest. The results are shown in **Figure 6**. The results showed that the maximum, minimum and variance of the multi-dimensional well-being composite index of the Dabie Mountain core area were respectively 0.6488, 0.1187 and 0.0959 in 2005 and 0.6841, 0.3147 and 0.0824 in 2020. The maximum and minimum values showed an upward trend, while the variation of variance decreased from 0.0959 to 0.0824, then the spatial difference in well-being level was equalizing. The results of the county analysis also showed that the multidimensional well-being comprehensive index of Jinzhai County in 2020 was 0.5205, which increased by 27.23% compared with 0.4091 in 2005. The quality of well-being and its spatial balanced development degree are the key to improving residents’ well-being (Wang et al., 2018). The analysis embodied that since the construction of the new countryside, the multi-dimensional comprehensive well-being index of the core area of Dabie Mountains has been on the rise as a whole, while the difference in the well-being of spatial units has been further narrowed, the well-being level of residents in the study area has been greatly improved during the last 15 years.

### 3.3 Geographical detection and optimization of spatial differentiation of coupling coordinated development degree

#### 3.3.1 Coupling coordination characteristics and geographical detection of ecosystem services and multidimensional well-being

**Figure 7:**
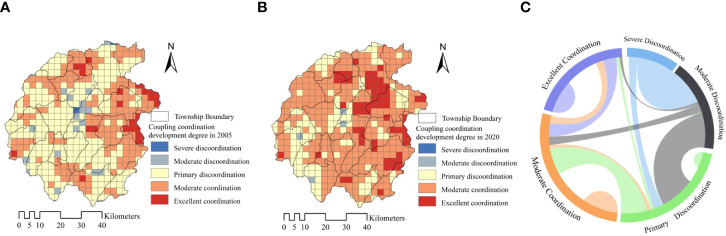
The distribution **(A, B)** and spatial transfer **(C)** of coupling coordination development degree.

The coupling coordinated development degree between the comprehensive index of ecosystem services and multidimensional well-being was calculated based on the coupling coordinated development degree, see formulas (9) ~ (10). The analysis showed that, in the core area of Dabie Mountains, there are 282 discoordinate grid units, accounting for 56.85%, and 214 coordination units, accounting for 43.15% in 2005. In 2020, the total number of discoordinate coupling units was 133, accounting for 26.81%, while the total number of coordination units was 363, accounting for 73.19%, as shown in **Table 3**. The spatial transfer analysis of coupling coordination development degree showed that, in 2005, 78.32% of the severely discoordinate areas turned to moderate coordination; 75.40% of the moderate discoordination turned into primary; 57.38% of primary discoordination turned to moderate coordination and 7.03% to excellent coordination; 16.34% of moderate coordination turned to excellent coordination, however, 6.94% still degenerated to primary discoordination; 28.60% of excellent coordination degenerated to moderate coordination, as shown in **Figure 7**. The analysis also indicated that the coupling discoordinate units showed a trend of sharply decreasing, with a drop of 30.04%, instead, the total number of coordination units has increased significantly, which presented a great development trend of the coupling coordination in ecosystem services and residents’ well-being.

**Table 3:** Degree of coordination development between ecosystem services and multidimensional well-being.

Type Year	Type of coupling coordination development									
	Severe Discoordination		Moderate Discoordination		Primary Discoordination		Moderate Coordination		Excellent Coordination	
	Number	Proportion	Number	Proportion	Number	Proportion	Number	Proportion	Number	Proportion
2005	4	0.81%	24	4.84%	254	51.20%	186	37.50%	28	5.65%
2020	0	0.00%	9	1.81%	124	25.00%	295	59.48%	68	13.71%
Variation	-4	-0.81%	-15	-3.03%	-130	-26.20%	+109	+21.98%	+40	+8.06%

In order to explore the spatial distribution and agglomeration characteristics of the coupling coordination development degree between ecosystem services and multidimensional well-being, this paper analyzed the global and local spatial autocorrelation by using the Geoda. The results indicated that the global spatial autocorrelation index Moran’s *I* of the coupling coordinated development degree between ecosystem services and multidimensional well-being index was greater than 0. Moran’s *I* in 2005 was 0.5670, while in 2020 Moran’s *I* decreased to 0.5511. The significance test showed that the *P-value* was far less than 0.01, thus the significance test at 1% level has passed, indicating that the coupling coordinated development degree between the ecosystem service index and the multi-dimensional well-being index had a significant feature of a spatial positive association in clustering, then the spatial agglomeration features showed a trend of multi-center distribution development, comparing 2020 with 2005.

In order to further explore the influence of spatial heterogeneity, taking 2020 as an example, factors such as nature, humanity and society were selected to conduct spatial heterogeneity geographic detection by using the GeoDetector. First, six factor layers of elevation (*X*_1_), slope (*X*_2_), NDVI (*X*_3_), habitat quality (*X*_4_), land use degree (*X*_5_) and human impact index (*X*_6_) were discretized and processed into type data based on ArcGIS 10.2. Then, the Geodetector tool was used to analyze the factor detection and interaction detection of the spatial differentiation of coupling coordinated development degree. The factor detector sorted these factors by q value as: habitat quality (*X*_4_) (0.2213) > human impact index (*X*_6_) (0.1690) > NDVI (*X*_3_) (0.1561) > land use degree (*X*_5_) (0.1512) >slope (*X*_2_) (0.0662) > elevation (*X*_1_) (0.0425). The results indicated that ecological quality,

vegetation cover and land use index factors had significant effects on the spatial differences of coupling coordination. Among them, the explanatory power of the habitat quality factor was more than 20%, which was the dominant control factor for the spatial difference of coupling coordination. Factor interaction detection can further evaluate whether the explanatory power of dependent variable Y will increase or decrease when different factors X act together (Wang and Xu, 2017). The analysis showed that a total of 7 groups of factor interaction detection *q* value reached above 0.6, all of which were nonlinear enhancement interaction types. The *q* value of the habitat quality (X4) ∩ human impact index (X6) was the highest (0.6489), which was the dominant factor of interaction detection (**Table 4**).

**Table 4:** The interaction detector for spatial differentiation of coupling coordination development.

Interaction Detection *q* value	Elevation (*X*_1_)	Slope (*X*_2_)	NDVI (*X*_3_)	Habitat Quality (*X*_4_)	Land Use Degree (*X*_5_)	Human Impact Index (*X*_6_)
Elevation (*X*_1_)	0.0425					
Slope (*X*_2_)	0.4741	0.0662				
NDVI(*X*_3_)	0.5809	0.5313	0.1561			
Habitat Quality (*X*_4_)	0.6465	0.6418	0.6404	0.2213		
Land Use Degree (*X*_5_)	0.5960	0.5066	0.5998	0.6470	0.1512	
Human Impact Index(*X*_6_)	0.6367	0.5179	0.6396	0.6489	0.2881	0.1690

Geographical detection analysis showed that the spatial differences of coupling coordinated between ecological and well-being in the core of Dabie Mountains, were affected by factors such as ecological environment, human activities and land use. A high-quality ecological environment and high-efficiency land use will further promote the improvement of coupling quality. In practice, the improvement of the ecological quality and land use level should be continuously strengthened. Especially in mountainous areas, the space for construction that is suitable for economic development is limited, thus it is necessary to rationally plan production, living and ecological development space according to local conditions, enhance land output benefits, strengthen habitat protection and ecological governance, and promote comprehensive benefits, so as to promote the synergistic improvement and “win-win” development of ecosystem services and residents’ well-being in the study area.

#### 3.3.2 Spatial coupling model of ecosystem services and multidimensional well-being

Strengthening the bivariate spatial autocorrelation analysis of the comprehensive index of ecosystem services and multidimensional well-being can further reveal the spatial coupling model between the two, which is helpful to explore the spatial agglomeration and mutual matching characteristics of the high value of “platform” and the low value of “depression” of ecological services and residents’ well-being. It also realizes the spatial visualization of the coupling model and has great significance for the collaborative improvement of ecosystem services and residents’ well-being level, the accurate identification of coupling quality, and the optimization regulation of “ecology-well-being”. In this paper, Geoda software was mainly used to analyze the features of the coupling model between the two. The calculation of the bivariate global spatial autocorrelation index showed that Moran’s *I* in 2005 and 2020 are -0.1768 and 0.0741 (*P* values are far less than 0.01, and they pass the significance test at 1% level). The research indicated that in 2005, there was a significant negative correlation between the comprehensive index of ecosystem services and multidimensional well-being in the study area, while in 2020, the two showed a positive correlation.

**Figure 8:**
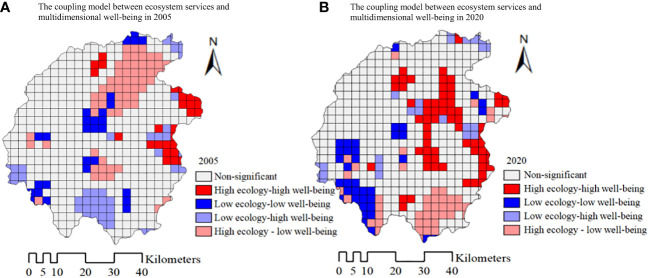
The coupling model between ecosystem services and multidimensional well-being in 2005 **(A)** and 2020 **(B)**.

Bivariate local spatial autocorrelation can provide support for further exploration of the coordinated development of ecological environment protection and human-land relationship (Zhang et al., 2018). The spatial distribution of four coupling models of ecosystem services and multidimensional well-being, named “high ecology - high well-being” HH, “high ecology - low well-being” HL, “low ecology - high well-being” LH, “low ecology - low well-being” LL can be reflected by the LISA Figure. Among them, HH and LL mean the high-level synergy and the low-level synergy with a positive correlation of ecosystem services and multidimensional well-being, while HL and LH mean the trade-off relationship between ecosystem services and multidimensional well-being with a negative correlation. As is shown in **Figure 8**, in 2005, there were 27 grid units in the “platform” area with high HH value, accounting for 5.44% of the total area, 29 grid units in the “depression” area with the LL model, accounting for 5.85% of the total area, while the HL and LH are 62 and 49. In 2020, with a little increase, there were 66 grid units with the HH model, accounting for 13.31% of the total area, 40 grid units with the LL model accounting for 8.06% of the total area, while the model of HL and LH showed a downward trend with the grid units of 45 and 25.

#### 3.3.3 Optimization strategies of ecosystem services and multidimensional well-being

The study showed that the number of synergistic coupling units of “high ecology - high well-being” were few, and most of them were “high ecology - low well-being” and “low ecology - high well-being” trade-off coupling models. During the past 15 years, the HH model area expanded to the central and southeastern areas, while the LL model area evolved from the central to the southwestern rural areas in the study area boundary. The spatial distribution of the LL model area had changed from scattered distribution to a few concentrated contiguous distribution models, forming an obvious “depression” in the core area of Dabie Mountains, while the HH model area further clustered into a “platform” in the core area of Dabie Mountains.

The results embodied that ecosystem services and multidimensional well-being are in large spatial differences in different areas and periods affected by the endowment of ecological resources, geographical location and living environment. Therefore, corresponding optimization strategies should be adopted for different types of areas according to local conditions so as to promote the coordinated improvement of ecological protection and residents’ well-being in the Dabie Mountain area. In consideration of “low ecology - high well-being” areas that mainly involve towns with high economic development and good transportation conditions, but land types there are mainly construction and agricultural, and the ecological environment is fragile. Therefore, in practice, the hotspots of human economic activities should enhance ecosystem service function through landscape pattern optimization, further strengthen ecological governance and restoration, improve ecological quality, and increase regional ecological service supply. While the “high ecology - low well-being” areas are covered with abundant vegetation resources and excellent ecological environment, mainly involving towns that have poor economic development level, traffic conditions, and infrastructure, it is imperative to intensify policy support and infrastructure investment, improve the residents’ production and living environment, establish diversified ecological compensation mechanism, increase the residents’ income, satisfaction, and the overall levels of welfare, etc., so as to achieve the “win-win” goal of ascension together. The “low ecology - low well-being” areas are the areas requiring focus and improvement, the ecological environment in these areas is often fragile, and ecological governance is difficult. Secondly, the construction of infrastructure and basic public service is relatively weak, the village collective economy lacks leading industries, and the income of residents is relatively low. Therefore, it is necessary to further explore the dynamic mechanism for the coordinated development of ecosystem services and residents’ well-being, strengthen the comprehensive ecological governance of land and space planning, establish and improve the alleviation mechanism for poverty. In addition, further identification of stakeholders related to ecosystem services and analysis of the regulatory mechanism of ecosystem services can provide an important basis for the management model of ecosystem service (Geng et al., 2021). To promote the optimization of regional ecosystem functions and the coordinated improvement of residents’ living standards, this is also the focus and difficulty of China’s work in the process of relative poverty monitoring, consolidating the achievements of poverty alleviation and connecting rural revitalization effectively. Finally, the “high ecology - high well-being” areas need to further optimize the function of the ecosystem and enhance resilience against ecological risks, meanwhile, relying on the advantages of rich ecological resources to cultivate ecological industries vigorously is a great way to improve high-quality development of the green economy. To a certain degree, the results can provide a reference for the diagnosis of coupling degree, accurate identification of spatial matching, and improvement of “win-win” strategies formulation synergistically for ecosystem services and the residents’ level of well-being in the Dabie Mountain area.

## 4 Discussion

Ecosystem services are the base of ecological security assessment and ecosystem regulation (Peng et al., 2018a), the synergistic development of ecosystem services and human well-being, with the complex interrelation, is a goal of ecosystem management (Zheng et al., 2013). Improving ecosystem services is also an effective way to increase human social welfare (Cao et al., 2018). For people in poor areas or vulnerable groups that depend on ecosystems, changes in ecosystem services have a great impact on human well-being (Wang et al., 2013). While the current researches on ecosystem services and multidimensional well-being have limitations such as unclear coupling mechanism, unclear regulation strategy, and lack of strategy design based on the analysis of mechanism. This paper explored the spatial-temporal evolution law and spatial heterogeneity from 2005 to 2020, revealed the coupling features and coupling model of the two quantitatively, and the results also corroborate the conclusion of the previous research on the coupling effect of man-land relationship (Pan et al., 2020; Zheng et al., 2020). For areas of the “low-low” synergistic coupling model and areas of “low ecology - high wellbeing”, as well as “high ecology - low well-being” trade-offs coupling models, we can further explore the stress causes and decoupling mechanism of the discoordination between ecological services and residents’ well-being, so as to provide management decision-making basis for solutions to the problem of regional coupling discoordination (Qiu et al., 2021). As the coupling mechanism between ecosystem services and human well-being is complex, although this study reveals the spatial-temporal difference characteristics of the coupling relationship between ecosystem services and human well-being, the multiscale coupling effect and multidimensional decoupling mechanism of ecosystem services and residents’ well-being in Dabie Mountains still need to be strengthened. Studies on the coupling of ecological processes and well-being show that changes in natural processes will affect the ecosystem services on which local populations depend, and that ecological conservation measures can affect the causal mechanisms of human well-being by altering ecosystem services (Yang et al., 2015). The “pattern-services-well-being” study shows that the correlation and the trade-offs between landscape patterns and ecosystem services have significant scale effects (Bai et al., 2020). The impact of ecosystem services on human well-being varies significantly across space, and the contribution of the same ecosystem service to the well-being of different groups varies significantly, so it is practically relevant to explore the subjective and objective dimensions of integrating human well-being, and to explore group differences in the impact of ecosystem services on human well-being based on multi-group analysis (Liu et al., 2022a). Furthermore, strengthening the sustainability conceptual cascade framework of “pattern-process-services” can help provide insight into the interactions between landscape patterns and ecological processes, as well as the complex linkages between ecological processes and ecosystem services that support human well-being, and is critical to promoting socio-ecological sustainability (Fu B. et al., 2022). As a static “ecology-well-being” assessment and coupling research process in this study, it is necessary to further explore multi-scenario simulation and other research (Chen et al., 2021), provide multi-scheme prediction and comparison for the research on synergistic improvement of ecological services and residents’ well-being, and further provide a theoretical basis for the exploration of the mechanism of overcoming difficulties in Dabie Mountain area and the research on “win-win” approaches of “ecology-well-being”.

## 5 Conclusion

Based on the InVEST model, the methods of spatial autocorrelation and coupling coordination, this paper discussed the spatiotemporal evolution, spatial heterogeneity and coupling models of ecosystem services and multidimensional well-being in the core area of Dabie Mountain from 2005 to 2020. The major results were shown as follows:

a) During 2005-2020, the analysis showed that there was a trend of “three items rise, and two items fall” in 5 typical ecosystem services, in detail, the annual growth rates of food supply, water yield and soil retention services were 2.03%, 8.17% and 6.04%, while the annual decrease rates of carbon sequestration and biodiversity services were 0.04% and 0.03%. Generally, ecosystem services showed an upward trend. In the past 15 years, the number of hot spots of multiple ecosystem services has increased, accounting for 10.09% of the total area, and the descending areas accounted for 10.71% of the area, the area that has not changed accounts for 79.20%, but little change as a whole.

b) The multidimensional well-being comprehensive index of the core area in Dabie Mountains in 2020 was 0.5205, which increased by 27.23% compared with 0.4091 in 2005, showing a high growth of residents’ well-being. The statistical analysis of spatial units in the study area indicates that in the past 15 years, the minimum and maximum values of the multidimensional comprehensive well-being index both showed an upward trend, and the variance change has decreased from 0.0959 to 0.0824. The results show that since the construction of the new countryside, the overall well-being of residents in the core area of Dabie Mountains has shown an upward trend, and the spatial differences of the well-being level in the study area have been further balanced.

c) During 2005-2020, the proportion of discoordinate coupling units between ecosystem services and residents’ well-being decreased from 56.85% in 2005 to 26.81% in 2020, while the number of coupling coordination units increased from 43.15% to 73.19%. The analysis of geographical detection showed that the habitat quality factor was the dominant controlling factor of coupling coordination spatial difference, meanwhile, the combined effect of habitat quality and human activities enhanced the impact of spatial differences, which was the dominant interaction factor of spatial difference enhancement. The bivariate correlation analysis showed that in the past 15 years, the area of the “high ecology - high well-being” coupling model in the core area of the Dabie Mountains has shown an upward trend, but the proportion was still small, while the proportion of the unbalanced correlation area was large.

## Data availability statement

The datasets presented in this article are not readily available because of restrictions in the use of questionnaire survey data. Requests to access the datasets should be directed to huangmuyi@ahjzu.edu.cn.

## Author contributions

MH and GZ wrote the original draft. QW contributed to editing. JW and WL performed formal analysis. SF, QK, and QG analyzed the data. QY performed supervision. All authors contributed to the article and approved the submitted version.
